# Spontaneous rectus sheath hematoma revealed by abdominal pain during pregnancy: A case report

**DOI:** 10.1016/j.ijscr.2024.109695

**Published:** 2024-04-24

**Authors:** Bacem Zaidi, Wael Gazzah, Mehdi Ben Saad, Sihem Sindi, Walid Maraach, Zied Mensi

**Affiliations:** a University of Sousse, Faculty of Medicine, Department of Surgery, Ibn El Jazzar Hospital, Kairouan, Tunisia; b University of Sousse, Faculty of Medicine, Department of Urology, Ibn El Jazzar Hospital, Kairouan, Tunisia; c University of Sousse, Faculty of Medicine, Department of Orthopedic Surgery, Ibn El Jazzar Hospital, Kairouan, Tunisia

**Keywords:** Rectus sheath hematoma, Abdominal pain, Pregnancy complications, Diagnostic imaging, Surgical procedures, operative, Conservative treatment

## Abstract

**Introduction and importance:**

Rectus sheath hematoma (RSH) is an uncommon but significant cause of acute abdominal pain in pregnancy, challenging in both diagnosis and treatment. It often arises from ruptured epigastric vessels and is associated with factors like anticoagulation therapy and previous abdominal surgery. Misdiagnosis, due to nonspecific symptoms, frequently leads to unnecessary surgeries, posing substantial risks to maternal and fetal health.

**Case presentation:**

We present a case of a 32-year-old multiparous woman at 31 weeks of gestation, experiencing right-sided abdominal pain and irregular contractions. With a history of four full-term deliveries and no recent trauma, her examination showed hemodynamic stability but featured pain upon movement and a notable blue discoloration in the left abdominal area. Moderate anemia was observed in lab tests. The diagnosis of RSH was confirmed via ultrasound and MRI. The treatment approach shifted from conservative to surgical due to deteriorating symptoms and falling hemoglobin levels.

**Clinical discussion:**

This case highlights the rarity and seriousness of RSH in pregnancy. Its non-specific symptoms complicate differential diagnosis, underscoring the need for prompt and precise diagnosis to avoid unwarranted surgical interventions. While conservative management is preferred in stable cases, surgical action is required in situations of instability or hematoma growth.

**Conclusion:**

RSH is a critical consideration in pregnant patients with acute abdominal pain. Early detection and tailored management are essential to mitigate surgical risks and ensure the safety of mother and child. This case reinforces the importance of vigilant and systematic patient evaluation to improve outcomes and minimize unnecessary surgical procedures.

## Highlights

• Rare case of spontaneous rectus sheath hematoma in a 32-year-old pregnant woman.
• Initial symptoms: irregular contractions, right-sided abdominal pain.
• Diagnosis via MRI after initial ultrasound showed no abnormalities.
• Treatment shifted to surgery due to worsening symptoms, falling hemoglobin.
• Case emphasizes the importance of considering rectus sheath hematoma in pregnancy abdominal pain diagnosis.

## 1 Introduction

Rectus sheath hematoma (RSH), although a rare cause of acute abdominal pain during pregnancy, presents significant clinical challenges. Although specific incidence rates during pregnancy are not well documented due to their rarity, studies in the general population estimate an occurrence of approximately 1.8 cases per 100,000, emphasizing their clinical significance when encountered [1]. It occurs due to a rupture in one of the epigastric vessels, leading to acute bleeding within the rectus sheath [2]. Risk factors associated with RSH include pregnancy, the use of anticoagulation therapy, a history of previous abdominal surgery, and trauma related to vigorous exercise or intense coughing [3]. In particular, the nonspecific presentation of RSH symptoms contributes to a high rate of misdiagnosis, estimated at approximately 93 % [4]. Such diagnostic inaccuracies can lead to unnecessary surgical interventions, such as emergent cesarean sections. These procedures increase the risk of morbidity and mortality, with reported rates of up to 13 % for mothers and 50 % for fetuses [5]. The decision to pursue a conservative or surgical treatment approach is contingent on a comprehensive assessment of various clinical and biological factors.

In this report, we examine the case of a multiparous woman, 30 weeks into gestation, who presented spontaneous RSH. This case report was carried out according to recent SCARE criteria [6].

## 2 Case presentation

Mrs. K, a 32-year-old woman with a gravidity of 6, parity of 3, and 2 prior spontaneous abortions, presented at 31 weeks of gestation experiencing irregular contractions and right-sided abdominal pain exacerbated by movement, palpation, and fetal movements. The workups following her previous spontaneous abortions did not indicate any clear etiological factors that could be associated with her current pregnancy complications. Until this presentation, her current pregnancy had been unremarkable. Her obstetric history was notable for four full-term deliveries, including three vaginal births and one via cesarean section.

The patient did not report having a history of recent trauma or episodes of intense coughing. On examination, she was hemodynamically stable, with vital signs within the normal range. Pain, located superiorly and laterally to the umbilicus, did not radiate but intensified with coughing and physical movements.

**Fig. 1:**
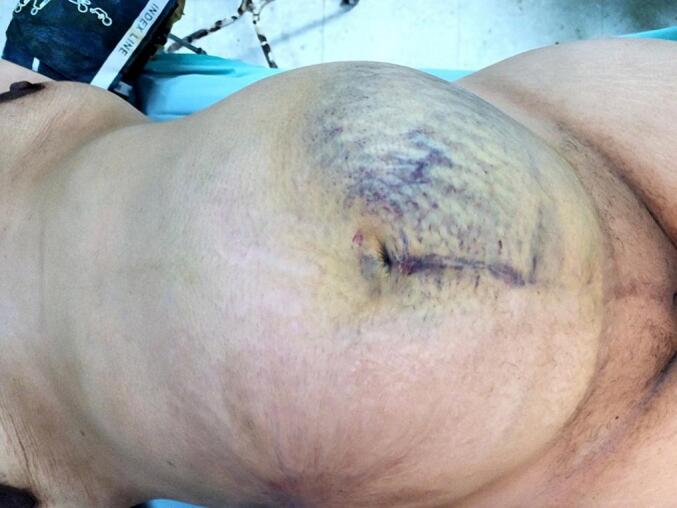
An image showing an abdominal hematoma on the left side.

Abdominal examination revealed a properly enlarged uterus for a gestation of 31 weeks. Moderate rigidity and tenderness was present, along with blue discoloration that covered the left abdominal quadrants and the right flank. No abdominal masses were palpable (Fig. 1). The vaginal examination did not show signs of bleeding and the cervix was closed. Fetal heart rate monitoring indicated a reassuring rate of 140 beats per minute, without observed uterine contractions.

Laboratory results indicated moderate anemia with a hematocrit (Ht) of 25.2 % and a hemoglobin (Hb) concentration of 8.4 g/dL. Platelet count was 132,000/mm^3^. The coagulation profiles, including the international normalized ratio (INR) at 1.01 and the activated partial thromboplastin time (APTT) at 30 s, were within normal limits. Leukocyte count and urine protein levels were also found to be normal.

Obstetric ultrasound demonstrated typical progression of pregnancy with fetal growth parameters within regular percentiles, and Doppler ultrasound findings were unremarkable. The placenta was placed posteriorly in the uterus, without evidence of retroplacental hematoma or signs indicative of acute placental abruption.

**Fig. 2:**
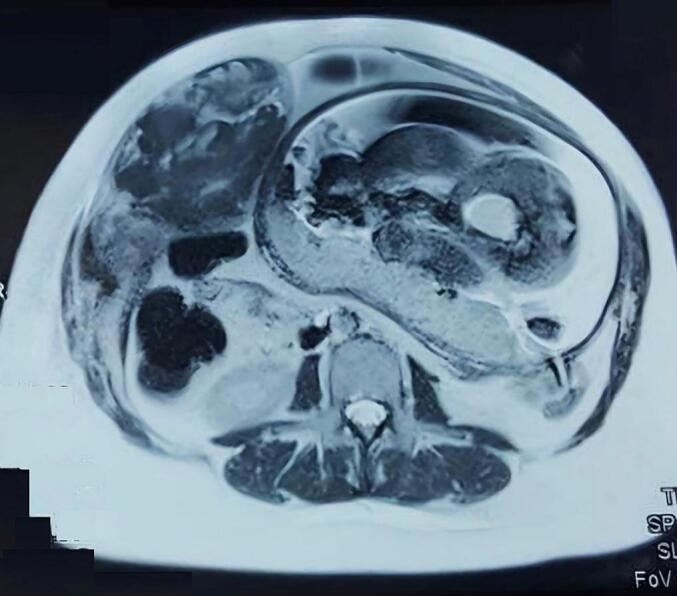
Magnetic resonance results showing hematoma.

An initial abdominal ultrasound did not reveal any abnormalities. However, 24 h after admission, the patient reported a marked increase in abdominal pain, to the extent that positional changes in the bed became intolerable. Consequently, a magnetic resonance imaging (MRI) scan was performed, which revealed a large suprafascial mass on the right side of the anterior abdominal wall, measuring approximately 11 × 6 × 20 cm (Fig. 2).

### 2.1 Diagnosis and therapeutic intervention

A diagnosis of Rectus sheath hematoma (RSH) was established and an initial conservative treatment approach was adopted. However, during the first 48 h of hospitalization, the patient's condition deteriorated, characterized by the onset of tachycardia and an intensification of her pain. During the same time, her hemoglobin levels decreased significantly to 6 g/dL. Given the abdominal findings, the progressive decrease in peripheral hemoglobin and the potential risk of fetal distress, betamethasone was administered to accelerate the maturation of the fetal lung. Subsequently, an urgent exploratory laparotomy was performed to evacuate the hematoma.

**Fig. 3:**
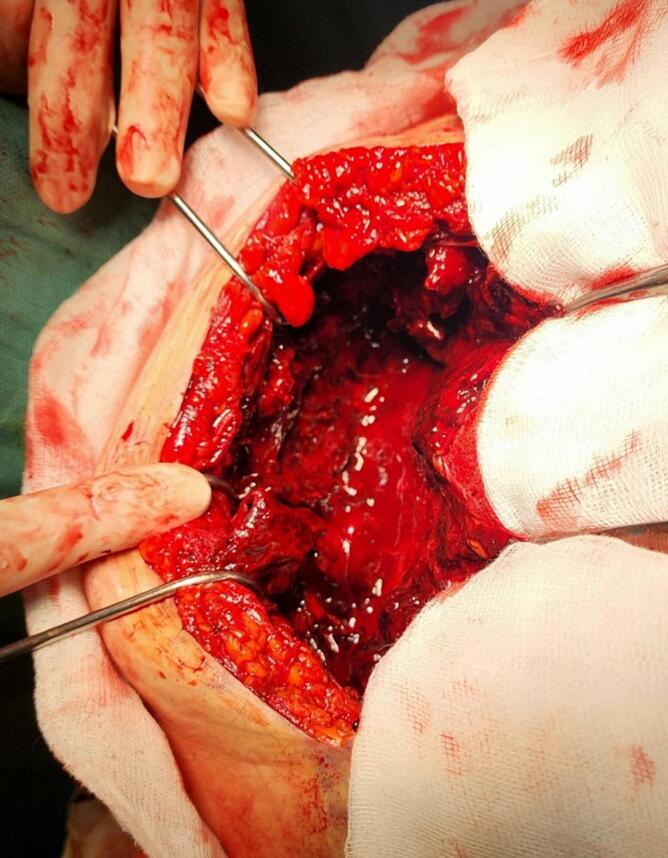
Peroperative view of the hematoma.

**Fig. 4:**
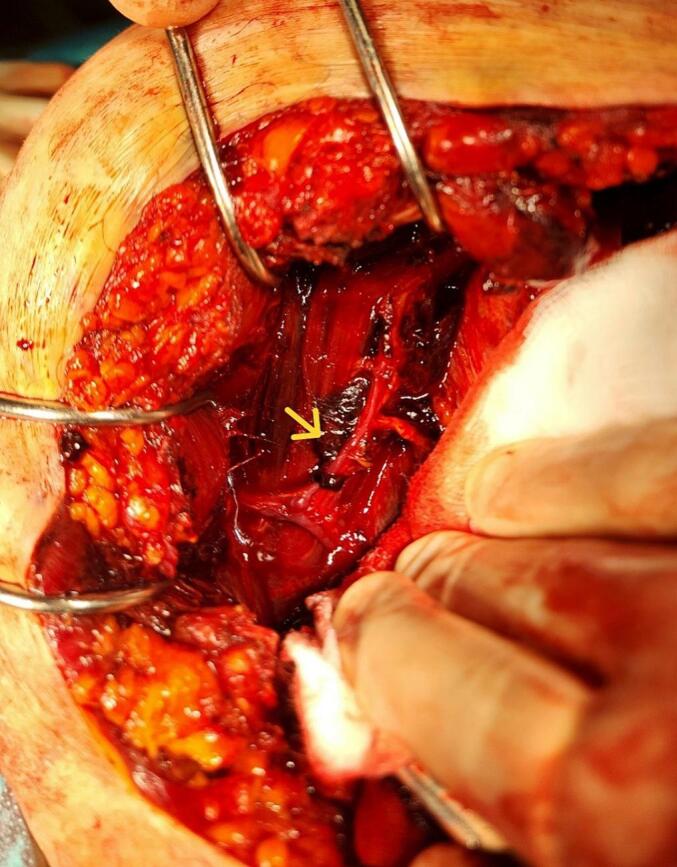
Branch of the epigastric causing bleeding.

Intraoperatively, a preperitoneal RSH was located adjacent to the right of the midline skin incision. The hematoma was meticulously evacuated from the sheath of the rectus abdominis muscle (Fig. 3). The branches of the epigastric vessel were identified and ligated (Fig. 4). A comprehensive examination of the uterus, fallopian tubes, ovaries, and upper abdominal region revealed no abnormalities.

### 2.2 Postoperative management and outcome

In the immediate postoperative phase, the patient received a transfusion of three units of packed red blood cells. Throughout this period, fetal monitoring indicated normal parameters. Post-transfusion, patient hemoglobin levels stabilized at 9 g/dL. She showed rapid recovery postoperatively and was subsequently discharged on the seventh day following surgery.

## 3 Discussion

Rectus sheath hematoma (RSH) represents a rare, yet critical condition, which mainly presents with acute abdominal pain and is potentially life-threatening. Its occurrence during pregnancy is rare, with limited cases documented in the literature. The diagnostic challenge posed by RSH is significant, as evidenced by its high misdiagnosis rate [7]. Misdiagnosis often leads to increased incidences of unnecessary exploratory laparotomies, cesarean deliveries, preterm births, and perinatal mortality [8]. Differential diagnosis in non-pregnant individuals includes a broad spectrum of conditions ranging from gastroenteritis to ovarian torsion, necessitating careful consideration [9].

The primary etiology of RSH involves trauma to the inferior epigastric artery. Contraction of the rectus abdominis muscle, which leads to tearing and tearing of the vessel, is a common mechanism [10]. Risk factors include conditions that elevate intra-abdominal pressure, such as severe coughing or vomiting, trauma, anticoagulation therapy, coagulopathy, vascular anomalies, female sex, previous abdominal surgery, and multiparity [11].

Clinical manifestations of RSH can be non-specific. Examination findings may include the Fothergill sign (a palpable, tender abdominal mass during rectus muscle contraction), Cullen's sign (periumbilical bruising), Carnett's sign (increased pain upon abdominal muscle tensing), or Turner's sign (flank bruising). Furthermore, low-grade fever and vomiting are sometimes reported [12].

Ultrasound, as a non-invasive and rapid diagnostic tool, is often used initially, especially in pregnant patients, to determine the size, location, and characteristics of the hematoma [13]. Magnetic resonance imaging (MRI) is valuable to differentiate chronic RSH from other masses of the anterior abdominal wall, although its diagnostic utility may be limited for acute hematomas within the initial 48-hour window [14].

Conservative management is the preferred approach for hemodynamically stable patients with non-expanding hematomas, who show mild symptoms and a clear diagnosis [2,7,9,15]. This strategy is recommended due to its effectiveness in managing symptoms while minimizing the risks associated with more invasive procedures. Conservative treatments may involve analgesia to manage pain, compression of the hematoma to reduce swelling, application of ice packs to decrease inflammation, and bed rest to prevent exacerbation of the hematoma. When necessary, more aggressive interventions such as intravenous fluid resuscitation are used to manage hemodynamic instability, and blood transfusion is considered if significant blood loss has occurred or if there is a marked decrease in hemoglobin levels. Typically, these hematomas resolve over several weeks [16], with ongoing monitoring to ensure that there are no changes in the patient's condition that would require a change in surgical intervention.

In contrast, surgical intervention becomes imperative in scenarios involving rupture, infection, or hemodynamic instability of the peritoneal hematoma that does not respond to initial fluid resuscitation. Surgical options include evacuation of the hematoma, vessel ligation, and closed suction drainage. An emerging alternative is percutaneous management, such as selective transcatheter arterial embolization, using thrombin or vessel coiling [2].

## 4 Conclusions

In summary, RSH represents a rare but potentially severe cause of abdominal pain that can cause significant bleeding. Its prompt identification is crucial and depends on a meticulous clinical assessment complemented by appropriate radiological investigations. Accurate diagnosis of RSH is paramount in avoiding unnecessary risks associated with surgical intervention, thereby safeguarding both maternal and fetal well-being.

This case underscores the importance of considering RSH in the differential diagnosis of acute abdominal pain during pregnancy. It also highlights the need for increased vigilance and a systematic approach to the evaluation of pregnant patients who present with such symptoms. Early and precise diagnosis, followed by customized treatment, can significantly improve outcomes and reduce the likelihood of unnecessary surgical procedures with their inherent risks.

## Patient perspective

The patient expressed relief and satisfaction with the treatments received, highlighting their effectiveness and the care provided by the medical team.

## Consent

Written informed consent was obtained from the patient for the publication of this case report and any accompanying images. A copy of the written consent form is available for review by the Editor-in-Chief of this journal upon request.

## Ethical approval

This study is exempt from ethical approval as per the policies of Ibn El Jazzar Hospital.

## Funding

No funding was received for conducting this study.

## Author contribution

All authors have contributed equally to the work reported in this manuscript, including the conception, design, execution, data acquisition, analysis and interpretation, and the drafting and revising of the manuscript for important intellectual content.

## Guarantor

Wael Gazzah.

## Conflict of interest statement

The authors declare that they have no conflicts of interest concerning this article.
